# Practices and Perceptions of Family-Centered Care: A Cross-Sectional Survey of Secondary School Athletic Trainers

**DOI:** 10.3390/ijerph20064942

**Published:** 2023-03-11

**Authors:** Zachary K. Winkelmann, Nancy A. Uriegas, James M. Mensch, Conner E. Montgomery, Toni M. Torres-McGehee

**Affiliations:** 1 Department of Exercise Science, University of South Carolina, Columbia, SC 29208, USA; 2 Honors College, University of South Carolina, Columbia, SC 29208, USA

**Keywords:** patient-centered care, sports medicine, communication, support system

## Abstract

Family-centered care (FCC) includes collaboration between families and healthcare providers, the creation of flexible policies, and the family taking an active role in the delivery of care. Secondary school athletic trainers provide care for underage patients in school-based health systems, making them responsible for maintaining communication with parents, guardians, and/or caregivers. This cross-sectional survey investigated the extent to which athletic trainers (n = 205) include aspects of FCC in their daily secondary school clinical practice (current practices = CP) and whether they believe that aspect of care is necessary for FCC to be provided in athletic training (perceived necessary = PN) in their everyday practice using the Family-Centered Care Questionnaire-Revised tool. The total mean score for the CP scale (mean = 26.83 ± 4.36) was significantly lower (*p* ≤ 0.01) than the PN scale (mean = 35.33 ± 4.17). All FCC subscales compared between CP and PN were significantly different (*p* ≤ 0.01), with each being of higher importance than CP in athletic training. Data analysis revealed four themes related to enhancing FCC in secondary schools: limited education and resources, staffing and space concerns, non-technical skills, and social determinants of health. Attention should be placed on developing resources and interventions for secondary school athletic trainers to collaboratively work with children and their support systems.

## 1. Introduction

Patient-centered care, which includes the healthcare provider being responsive to and respectful of the patient’s values and needs in their clinical care, has been widely studied across healthcare professions, specifically in athletic training [1,2]. Athletic training is a healthcare profession that provides medical services to individuals involved in sports, physical activity, and work environments. The practice of athletic training involves five domains, including (1) risk reduction, wellness, and health literacy; (2) assessment, evaluation, and diagnosis; (3) critical incident management; (4) therapeutic intervention; and (5) healthcare administration and professional responsibility [3]. Athletic trainers are educated at the postbaccalaureate level and must pass a board exam for national certification to practice clinically. The role of the athletic trainer is to work collaboratively to ensure patient-centered medical services [3]. In a patient-centered environment, an individual should expect their provider to invite them to ask questions and make sure they are in full collaboration with their treatment plan decisions [4]. Previous research specific to athletic training identified collegiate student-athletes who believed empathy was important and that with patient-centered communication, there would be a solid relationship and result in better outcomes [5]. Moreover, most collegiate student-athletes viewed their athletic trainers to be patient-centered in terms of individualized care, the patient being a priority to them, and they felt they received the best options possible [4]. However, goal setting continues to emerge as a limitation of communication strategies deployed by the athletic trainer [4,6].

While most encounters involve patient-centered care through the lens of working with adults, the situation faced by athletic trainers in secondary schools is unique. In most patient encounters, secondary school athletic trainers typically care for patients under the age of 18 in a school-based health system and are tasked with collaborating with other healthcare professionals, but most importantly, are responsible for maintaining constant communication with the parents, guardians, or caregivers of minors [7]. Due to the age group of these patients, there must be strong communication with the family on the care and treatment these student-athletes are receiving. It is the responsibility of the athletic trainer in secondary schools caring for adolescent athletes to be familiar with and comply with state laws and regulations specific to the consent authority. While it is required by law to communicate with a parent/guardian when treating minors, parent engagement with the healthcare provider may differ. The literature supports that parents value patient-centered care, also referred to as child-centered care in this case, and the ability to provide input when dealing with their children’s health [8]. The inclusion of strong communication and connection to the families when talking about adolescent healthcare is known as family-centered care (FCC) [9]. When talking about this form of care, it can be described as including “information sharing, partnering, respect, and negotiation” that prioritizes the engagement of the patient’s family in the shared decision-making process [9]. Families are typically important support systems for children. The family wants to be told information on their child’s injury or illness, the course of action and treatment, and the prognosis. Without communication on these things, families could be left out of key decisions and possibly lose trust in the healthcare provider. The family not only serves as a patient advocate in the process but also provides financial assistance and emotional support outside of the healthcare facility [10]. Previous research identified parents were aware of the training and knowledge of athletic trainers and felt having a provider on site was a key to safety [11]. Engaging with parents, siblings, and other family members in the healthcare process for an adolescent student-athlete allows for the athletic trainer to have insight into their patient’s specific social determinants of health, preferences, and cultural considerations necessary to individualize the care process [12].

However, the concept of FCC, whereby the family takes an active role in the planning, implementation, and evaluation of the care and has a say in the care of the child as much as a health professional, has not been examined in athletic trainers. The aim of FCC is to maintain the connections between the underage patient and their family, to prioritize the effectiveness in the care of the child, and to prevent or minimize the negative effects of illness, injury, or hospitalization. Research indicates the universal components of FCC include collaboration between family members and healthcare providers, the creation of flexible policies and procedures, and the need for patient and family education [13]. The skills and behaviors align with the expected contributions of an athletic trainer providing care in middle and high school settings. Therefore, the purpose of this study was to describe the perceptions and practices of FCC care across secondary school athletic trainers.

## 2. Materials and Methods

We used a cross-sectional survey design to explore secondary school athletic trainers’ practices and perceptions of FCC. We used the Strengthening the Reporting of Observational Studies in Epidemiology (STROBE) Statement checklist for cross-sectional studies to guide the presentation of this manuscript. This study was conducted according to the guidelines of the Declaration of Helsinki and deemed exempt from approval by the Institutional Review Board of the University of South Carolina (Submission: Pro00121032; Approval Date: 23 May 2022).

### 2.1. Instrument

To explore FCC, the research team used the content-validated and reliable Family-Centered Care Questionnaire-Revised (FCCQ-R) [14,15]. In addition, the survey contained five demographic questions about the participant’s age, gender, years of credentialed experience as an athletic trainer, the highest level of education, and the type of secondary school they work in (e.g., private school, public school, or other). The FCCQ-R contains 45 items surrounding one’s beliefs about what FCC is or is not. Each item was assessed by the participant on the two FCCQ-R scales, which include the current practiced (CP) scale and the perceived necessary (PN) scale. The two FCCQ-R scales are further comprised of nine subscales, including (1) family is the constant, (2) parent/professional collaboration, (3) recognizing family individuality, (4) sharing information with parents, (5) developmental needs, (6) parent-to-parent support, (7) emotional and financial support, (8) design of healthcare system, and (9) emotional support for staff. Each item was assessed on both scales using a 5-point Likert scale from 1 = strongly disagree to 5 = strongly agree, with a higher number indicating a higher perception or practice of the item specific to FCC. In addition, the survey included an open-ended question where participants were able to write additional comments or suggestions of what they believe is needed to enhance family-centered care.

Due to the nature of the FCCQ-R items being written about hospital care with words such as admission and facilities, the research team for this study engaged in a minor revision process before data collection. In total, 13 of the 45 items in the tool required grammatical changes. A panel of 3 athletic trainers with experience working in the secondary school setting, as well as doctoral-level training in research, was convened to content validate the changes. The primary investigator made the initial edits to the FCCQ-R items and sent the revised tool via e-mail to the expert panel for feedback. After feedback was collected, edits were made to the wording only on the changes (for example, hospital to athletic training facility). Following the edits, a content validation index (CVI) was performed for the clarity and relevance of the proposed changes [16]. This resulted in a CVI relevancy average score of 0.961/1. In addition, a clarity average score of 0.955/1 was also achieved. With both CVI scores greater than or equal to 0.9, the tool modifications were considered to be content validated [16]. We identified an overall total Cronbach’s alpha for the CP: α = 0.945 and PN: α = 0.946 scales demonstrating high reliability. Table 1 provides the full-scale and subscale reliability assessment.

### 2.2. Participants

This study aimed to explore FCC with athletic trainers working in the secondary school setting across the United States. We used G*Power software (version 3.1.9.4; Franz Faul, Universität Kiel, Kiel, Germany) to explore an appropriate sample size for the study. Using an α = 0.05 and a moderate effect size (0.3), the power calculation indicated a sample size of 132 participants was needed for an estimated power of 0.95.

To do so, we recruited the entire sample (n = 7695) of eligible individuals from this job setting that were also members of the National Athletic Trainers’ Association (NATA) and opted into the research participation database. A total of 504 athletic trainers accessed the survey (response rate = 6.5%), and 305 individuals started the survey. Overall, 205 athletic trainers in the secondary school setting (men: n = 91, women: n = 113, preferred not to say: n = 1; age range: 18 to over 65 years) completed the survey yielding a 67.2% (n = 205/305) completion rate. The data from complete responses were used for the analysis. Full demographic information for the participants is presented in Table 2.

### 2.3. Procedures

The recruitment e-mail was sent by the NATA research database describing the study and providing directions for completing the survey, as well as a direct link to the survey via a secure, web-based system (Qualtrics, Provo, UT, USA). Data collection began in May 2022 with reminder e-mails sent every week for four weeks (June 2022) to unfinished respondents. Due to the timing of the study (May–June), which aligned with the end of the traditional secondary school academic calendar, we opted to complete an additional 4 weeks of data collection using the same sample of unfinished respondents with reminder e-mails beginning again in September 2022 for four weeks ending in October 2022.

After opening the survey link, participants were provided with an invitation to participate and the ability to indicate their willingness to engage in the study. Individuals that did not consent to participate (n = 20) and/or indicated they were not currently employed as an athletic trainer in the secondary school setting (n = 18) were excluded from the study. For those that met inclusion criteria and after consenting to participate, the participants were directed to the survey items, answering only the questions they wanted to, and could close the browser at any time. Participants completed the 45-item FCCQ-R instrument, one open-ended item about enhancing FCC, and five demographic questions. Specific directions and definitions were provided to the participant on the FCCQ-R. Participants were instructed to consider the extent they include this aspect of care in their everyday work at their secondary school (CP scale) and whether they believe this aspect of care is necessary for FCC to be provided in athletic training (PN scale). The questions on the tool also identified staff and family, which was each operationally defined in the survey with staff meaning “any health professional who provides direct care for children and their families, for example: school nurse, athletic trainer, social worker, clergy, psychologist, physician, etc.” and “family refers to parents, guardians, grandparents, and siblings, and any family friend who is significantly involved with the child’s everyday life or care” [14,15].

### 2.4. Data Analysis

The data were collected and stored on the web-based platform. The completed survey responses were downloaded to SPSS (IBM Corp., IBM SPSS Statistics for Windows Version 28, Armonk, NY, USA) for statistical analyses. The data were first scored by adding scores for each item on the scales for each of the practice and perception to calculate a mean score. A total CP and PN scale score was also calculated by summing the mean for each of the 9 subscales, which were used to compare perception and practice. Scales were included if all or all but 1 item was answered for the questions within that scale. Means were calculated for the 2 scales and 9 subscales. The data were analyzed using paired samples *t*-tests to explore differences between the CP and PN scales.

Finally, of the 205 participants, 44 secondary school athletic trainers responded to the one open-ended question in the survey in response to “please write any additional comments or suggestions of what is needed to enhance family-centered care.” Qualitative data were analyzed using an inductive process to examine trends in participant responses. Trustworthiness was achieved through researcher triangulation, where two members (Z.K.W., J.M.M.) of the research team coded the data and identified main themes separately before comparing findings and coming to a consensus. A strength of this design is that qualitative data can be used to provide richness and complement the quantitative findings. The data are presented as extracted quotes that best characterize each theme.

## 3. Results

### 3.1. Practices and Perceptions

Mean scores and paired samples *t*-tests on the practice and perception scales of FCC are presented in Table 3. The total mean score for the CP scale (mean = 26.83 ± 4.36) was significantly lower (*p* ≤ 0.01) than the PN scale (mean = 35.33 ± 4.17). The parent-to-parent support collaboration subscale had both the most practiced (mean = 3.93 ± 0.85) and highest perceived need (mean = 4.61 ± 0.73). In addition, the developmental needs subscale had both the lowest score for CP (mean = 2.62 ± 0.79) and was considered by secondary school athletic trainers as the least necessary element of FCC (mean = 3.23 ± 0.76). The smallest subscale discrepancy between CP and PN was family is the constant meaning their perceptions and practice closely align. All FCC subscales compared between CP and PN were significantly different (*p* ≤ 0.01), with each being of higher importance than CP in athletic training.

### 3.2. Open-Ended Responses

Data analysis revealed four themes related to enhancing FCC in secondary schools: limited education and resources, staffing and space concerns, non-technical skills, and social determinants of health. Athletic trainers consistently described not having the appropriate education or resources to integrate FCC appropriately into their setting. They also highlighted staffing and space concerns that would not allow them to effectively communicate and connect to families when they have so many athletes and so few staff members. The third theme suggested that to adopt FCC correctly, athletic trainers need to have or acquire non-technical skills such as communication, establishing trust, and developing relationships. The final theme from the data indicated social determinants of health play an important role and must be considered when integrating FCC into secondary schools. Table 4 provides extracted quotes from the data that best describe the associated theme.

## 4. Discussion

The present study aimed to describe the perceptions and practices of FCC across secondary athletic trainers, making this the first study to explore this concept in this profession. In athletic training, the Board of Certification Standards of Professional Practice states athletic trainers must inform “the parent/guardian of a minor patient of any risks involved in the treatment plan” and “develops and maintains a relationship of trust and confidence with the parent/guardian of a minor patient” [17]. The data from our study suggest athletic trainers feel they are practicing FCC, with the exception of “developmental needs.” However, there were significant differences between their practices and perceptions of FCC. These findings are consistent with previous studies conducted across other healthcare settings, such as nursing and neonatal intensive care units [14,18,19]. In addition, research has assessed the attitudes toward FCC among physical therapists, professionals with similar skill sets as athletic trainers, in the United States [20]. The highest scores observed in that study included the respectful and supportive care subscale, which can be described as making parents feel respected as individuals, equals, and experts [20]. Overall, there are several well-documented benefits of FCC in other peer healthcare professions, and we believe this study could help to bridge the gap between the FCC principles in sports medicine.

### 4.1. Parent-to-Parent Support

At the time of injury, secondary school athletes reflected that they worried, as well as their family and friends, about the severity of their injury [21]. In addition, previous research has identified families are at risk of developing mental health challenges, such as post-traumatic stress disorder, after their child sustains an injury [22]. This sense of worry can be reduced or eliminated through parent-to-parent support groups. As originally proposed by the Association for the Care of Children’s Health [23], parent-to-parent support means assistance provided by a parent who may be facing similar challenges and who can understand what one is going through. Parents have expressed this as a key element of FCC [23]. Parents feel the power of shared experiences allows them to empathize and be validated when facing difficult challenges with the health of their children. Furthermore, dating back to 1986, Minna Nathanson outlined three important functions of parent-to-parent support, which include (1) mutual support and friendship, (2) information gathering and sharing, and (3) improving the system [24]. Overall, parents want the opportunity to help each other identify information to navigate the hardships associated with caring for their children and develop support systems along the way.

Athletic trainers reported parent-to-parent support as the highest perceived and most frequently practiced element of FCC. Interestingly, this element is not ranked highly across other healthcare professions for both perception and practice [18,19,25,26,27,28,29,30]. Athletic trainers reported practicing parent-to-parent support (mean = 3.93 ± 0.85) more frequently as compared to other professions, with mean scores ranging from 2.38 to 3.55 [18,19,26,28,29,31]. Similarly, athletic trainers perceived this as a necessary element more than other healthcare professionals. To our knowledge, the studies conducted by Franck et al. and Petersen et al. are the only studies conducted in the U.S. to date that utilized the FCCQ-R [18,25]. Participants in these studies reported similar perceptions as athletic trainers on the parent-to-parent scale, with scores of 4.23 and 4.04. It can be possible that healthcare professionals in the U.S. understand and value the shared experiences that parents have with regard to the care of their children and may promote it more than in other countries. It is of future interest to explore how athletic trainers coordinate and provide parent-to-parent support in the secondary school setting.

### 4.2. Developmental Needs

Across athletic trainers, the least practiced and lowest perceived necessary element of FCC was developmental needs. In its original form, the incorporation of the developmental needs of children, adolescents, and their families into the healthcare systems was primarily intended to serve individuals with disabilities [23]. The literature exploring FCC has predominately been conducted across nurses in pediatric or neonatal hospital units, where patients may face significant challenges with regard to growing up with disabilities and how to educate patients to be self-sufficient and independent [18,25,30]. Findings indicate that most healthcare professionals practice this element of FCC and perceive it as necessary, with mean scores as high as 4.44 [28]. In comparison, athletic trainers’ perception of developmental needs was 3.23. Secondary school athletic trainers do not typically face these developmental challenges, as they work with a population who is highly active and engaged in intense physical activity, and a possible explanation to why they did not perceive developmental needs as highly as other elements of FCC.

Following an injury or illness, a patient may experience emotional and social developmental challenges that affect their interest and motivation. While the patients’ athletic trainers typically care for primarily able-bodied children, a focus needs to be placed on the emotional and social components when the child experiences an injury. Practicing and incorporating this element of FCC may be beneficial when caring for patients instead of a compartmentalized approach that focuses on the injury separate from the emotional and social aspects. To do so, we recommend athletic trainers approach patient care using the World Health Organization International Classification of Functioning, Disability, and Health (ICF) disablement model framework [32]. The ICF model allows the clinician to explore other areas outside of the health condition, which could highlight developmental needs such as body functions, activity limitations, participation restrictions, environmental factors, and personal factors [32].

### 4.3. Family Is the Constant

Athletic trainers provide services in secondary school after obtaining parental consent at the onset of the school year rather than at each patient encounter [33]. This could be detrimental to the relationship and promoting FCC as it limits the connectivity between the parties, which reinforces the lack of knowledge and limited experiences for parents to meet and understand the role of the athletic trainer [34]. Athletic trainers recognize that a relationship with parents is important and could serve as a barrier to providing FCC if not present. To implement FCC, it is imperative athletic trainers acknowledge that *family is the constant* construct. This means that as the adolescent discontinues sport participation, recovers from their injury or illness, and/or graduates from secondary school, their support system (i.e., parents, guardians, siblings) will continue while the athletic trainer’s role is temporary. While not the highest practiced element of FCC, the construct that the *family is the constant* had the lowest discrepancy between the CP and PN scales. Across the literature, this theme does not seem to be uncommon, with other health professions stating the same sentiment. In fact, the family is the constant scale is commonly the highest practiced FCC [14,18,19,25,26]. Similarly, the implementation gap between perceptions and practice of this element of FCC has had the smallest differences across other studies [14,18,25,29]. Altogether, the “family as a constant” is a theme that health professionals, including athletic trainers, perceive as important when practicing FCC.

Furthermore, it can be assumed that athletic trainers, in general, believe proper communication with the family and a good trusting relationship is vital for ensuring quality FCC. Unfortunately, athletic trainers in the secondary school setting cited parents as both the most common job-related stressor and source of conflict [35]. There is a disconnect between the clinician-centered care approach from the athletic trainer resulting in an uninformed parent, a confused child, and a result-oriented athletic trainer. We recommend athletic trainers change their perspective on the role of the parent/family. The theory of social capital suggests leveraging the family as a resource to build relationships focused on trust, safety, and social cohesion to influence the sharing of information, increase health outcomes, and accomplish shared goals [36]. In the case of a patient–provider relationship, the athletic trainer can build trusting relationships with the families to improve the minor’s health. A key strategy to incorporate is creating multiple communication options that meet the needs of the parents in terms of time and accessibility and avoiding medical jargon. The integration of FCC into clinical practice may increase the short-term burden of stress and conflict but ultimately should promote a common goal between the family and the athletic trainer. However, the “best interest” of the child held by the family and the athletic trainer may differ, resulting in conflict, power imbalances, and expert tension [37]. A natural first step in changing the culture of healthcare for athletic trainers in the secondary school setting would be to explore the perception and reputation held by the student-athletes, family members, and personnel. The data should explore the current perceived training, skills, opportunities, and areas for necessary improvement. The commitment to engaging and embracing the family throughout the process could have positive, long-term benefits for addressing the healthcare of the adolescent.

In addition, previous research has identified parents need greater education on issues that they can deem important for their children’s health [38]. It is critical athletic trainers prioritize family education, as well as patient education, to encourage collaboration throughout the care plan. In doing so, the athletic trainer should explore the parent’s health literacy in the care decision-making process and provide education and options individualized to the family [39]. Specifically, there is a need to encourage home care management facilitated by the parents or guardians. The active engagement of the family in the therapeutic intervention process could improve buy-in from both the patient and family and alleviate the perceived stressors and conflicts.

### 4.4. Exploring the Qualitative Findings

We identified four themes from the open-ended responses related to an athletic trainer’s ability to effectively implement an FCC approach in secondary schools. These themes are consistently noted in athletic training and include limited education and resources, staffing and space concerns, non-technical skills, and social determinants of health. Staffing concerns for secondary school athletic trainers are not a new issue and can potentially affect the overall healthcare of athletes. McGuine et al. categorized over 2400 athletes based on athletic trainer availability and found that those with more access to an athletic trainer were positively influenced to report a sports-related concussion [40]. In addition, subsequent post-concussion management was also enhanced by the presence of an athletic trainer. The problem identified in our research (Table 4) is that there is a shortage of athletic trainers employed in the secondary school setting for a variety of different reasons. Research in secondary schools suggests athletic directors and principals would like to employ athletic trainers in their schools, but they indicate several barriers [41]. Athletic directors identify budget concerns, rural locations not close in proximity to hospitals/clinics, and misconceptions about the role of an athletic trainer as factors that contribute to their inability to hire athletic trainers [41]. A recent study indicated 66% of secondary schools in the U.S. have access to athletic training services [42]. The lack of adequate staffing in secondary schools is problematic for athletic trainers in providing quality healthcare, including FCC and other essential healthcare practices.

Even when secondary schools are able to hire athletic trainers, our findings suggest they are inadequately trained or provided with the education and skills to implement FCC within their clinical practice. Athletic trainers see the need for continuing education to advance their practice [43], but evidence suggests there are barriers such as time, cost, and associated travel which ultimately deter the completion of continuing professional development [44,45]. Professional athletic training education programs should incorporate the tenants of FCC instead of waiting for an individual to self-select continuing education opportunities on FCC. There is a need for professional education, as well as residency and fellowship experiences in athletic training, to align with the Accreditation Council for Graduate Medical Education (ACGME) standards for patient- and family-centered care [46]. The Athletic Training Milestones have been established as a guiding assessment framework that mirrors the ACGME standards [47]. While our study focused on the specific FCC scales, the ACGME Milestones describe general interpersonal and communication skills through the competency framework. We recommend future graduates of professional athletic training programs and all current practicing athletic trainers be able to (1) establish and maintain a therapeutic relationship using effective communication behaviors in challenging patient encounters and (2) identify complex barriers to effective communication, including personal bias [48].

Findings from this study suggest athletic trainers need more formal training and education on FCC, as well as a better understanding of non-technical skills to integrate concepts of FCC into clinical practice. Participants from this study consistently spoke about the need for athletic trainers to use non-technical skills such as building trust, establishing relationships, and communicating appropriately with families to truly integrate FCC. A parent’s perception of an athletic trainer’s skillset is vital to the success of FCC. Previous research in secondary schools indicated that 55% of parents did not always trust the athletic trainer’s opinion [49]. Weitzel et al. demonstrated that parents do value athletic trainers as important members of the secondary school healthcare team but also found that parents have varying perceptions of athletic trainers’ skill levels based on previous experiences [34]. The interactions between the athletic trainer and family are critical and ultimately determine if the athletic trainer will be able to successfully implement FCC.

The final theme to emerge from the qualitative data was the impact social determinants of health might play in implementing FCC. Athletic trainers indicated that the social determinants of health must be considered when trying to use an FCC approach, specifically in the secondary school setting. Research in athletic training has highlighted that athletic trainers can have a positive outcome in reducing negative social determinants of health experiences [50]. A qualitative research study by Hernandez et al. examined secondary school athletic trainers’ experiences with providing healthcare to low socioeconomic status patient populations [51]. Results were consistent with participant comments from our study, which both suggested athletic trainers are in a good position to support and advocate for low socioeconomic populations. Participants’ comments from this study also corroborated other findings from the qualitative analysis whereby participants shared that continuing professional development was needed and suggested that they were ill-equipped to navigate socioeconomic status challenges as they delivered care to patients in secondary schools. If athletic trainers in secondary schools want to incorporate FCC into their practice, more resources, including education (formal and informal) and the number of staff, will most certainly need to be provided. Many of the non-technical skills needed to care for patients will most likely be learned over time through experiences and interactions with different patients; however, there is a need for continuing professional development related to inclusion and equity for complex family dynamics. Athletic trainers will also need to gain a better understanding of the social determinants of health as they use FCC in their practice, as well as consider screening for these social factors to create individualized care plans and resources specific to the family [52]. Athletic training education programs should focus on exposing students, both clinically and didactically, to the challenges identified in this research study to incorporate FCC into their practice.

### 4.5. Limitations and Future Research

This study has potential limitations. While over 66% of secondary schools employ an athletic trainer, a small portion participated in this study, making the primary limitation the generalization of these results. Survey research in athletic training historically has suffered from small sample sizes, but the data from our study aligned with the sample size power analysis and should be shared to encourage future efforts surrounding FCC. We suggest attention should be placed on developing interventions and providing resources for secondary school athletic trainers to collaboratively work with patients and families in their setting. While the main concern of staffing and space cannot be directly addressed from the findings, we suggest formal education on delivering FCC is a key first step for stakeholder buy-in. Finally, future research should explore parent and family perceptions of their athletic trainers regarding the delivery of child- and family-centered care.

## 5. Conclusions

Athletic trainers in the secondary school setting are vital components of the healthcare team for middle and high school athletes in the US. Overall, most athletic trainers self-reported that they are performing all FCC aspects significantly less in their daily practice than they perceive necessary. The participants reported the FCC components were needed more in daily clinical practice than what was currently happening. In this setting, athletic trainers may feel compromised by time to educate, inform, and collaborate with the family to provide support. Participants noted barriers and concerns to FCC that may explain the significant differences in perception and practice.

## Figures and Tables

**Table 1:** Reliability Assessment.

Subscales	# of Items	CP α	PN α
Family is the constant	3	0.503	0.445
Parent and professional collaboration	6	0.656	0.673
Recognizing family individuality	5	0.799	0.838
Sharing information with parents	5	0.713	0.750
Developmental needs	4	0.784	0.804
Parent-to-parent support	5	0.707	0.695
Emotional and financial support for families	4	0.704	0.764
Design of healthcare system	7	0.804	0.871
Emotional support for staff	6	0.851	0.816
Total Scale	45	0.945	0.946

Note: FCCQ-R = Family-Centered Care Questionnaire-Revised; α = Cronbach’s alpha.

**Table 2:** Participant Demographics and Professional Information (reported in % and sample size).

Age Group		% (n)
	18–24 Years Old	7.3 (16)
	25–34 Years Old	35.2 (77)
	35–44 Years Old	17.4 (38)
	45–54 Years Old	20.1 (44)
	55–64 Years Old	11.0 (24)
	65+ Years Old	2.7 (6)
Gender		
	Men	41.6 (91)
	Women	51.6 (113)
	Non-Binary/Third Gender	0 (0)
	Prefer to Self-Describe	0 (0)
	Prefer Not to Say	0.5 (1)
Education		
	Bachelor’s	21.0 (46)
	Professional Master’s	22.4 (49)
	Post-Professional Master’s	43.4 (95)
	Clinical Doctorate	5.0 (11)
	Academic Doctorate	2.0 (4)
Employment Position		
	Public Secondary School Full Time	65.3 (143)
	Private Secondary School Full Time	16.0 (35)
	Public Secondary School Part Time	10.0 (22)
	Private Secondary School Part Time	2.3 (5)
Years of Professional Experience		
	<5 Years	29.7 (65)
	6–10 Years	13.7 (30)
	11–15 Years	17.4 (38)
	16–20 Years	9.1 (20)
	21–25 Years	23.3 (51)
	26+ Years	0 (0)

**Table 3:** Differences between athletic trainers’ practices and perceptions of FCC.

Subscales	Scales			
	Current Practices	Necessary Perceptions	*t*-Statistic	*p*-Value
Family is the constant	3.76 (0.61)	3.92 (0.58)	−5.11	<0.01
Parent and professional collaboration	3.06 (0.53)	3.40 (0.56)	−10.50	<0.01
Recognizing family individuality	3.87 (0.66)	4.26 (0.52)	−11.65	<0.01
Sharing information with parents	3.73 (0.66)	4.26 (0.50)	−13.21	<0.01
Developmental needs	2.62 (0.79)	3.23 (0.76)	−13.77	<0.01
Parent-to-parent support	3.93 (0.85)	4.61 (0.73)	−13.83	<0.01
Emotional and financial support for families	3.75 (0.68)	4.09 (0.62)	−9.50	<0.01
Design of healthcare system	3.04 (0.71)	3.79 (0.62)	−16.72	<0.01
Emotional support for staff	3.01 (0.93)	3.84 (0.04)	−15.89	<0.01
Total Scale	26.83 (4.36)	35.33 (4.17)	−28.90	<0.01

**Table 4:** Supporting Quotes.

Theme	Supporting Quotes
**Limited education and resources**	“I have very little knowledge and training about [FCC].” “Continuing education programs would definitely help. Most of the time in the secondary school situation, one, and if you’re fortunate enough, two athletic trainers are the healthcare specialists for 500+ athletes within a multitude of sports and different levels of competition. You take care of everyone you can and refer to “outside” specialists on an “as needed” basis. You always work towards open communication among all parties concerned and sometimes it works wonderfully well; other times, not so well. Our intention is to provide the best healthcare possible to our athletes. Possibly adopting a family-centered care approach may help us achieve a greater level of care if it can be done efficiently and be cost effective.” “Helping guardians understand the implications for going against medical care is something missed in education and continuing education—this is difficult to navigate but important to the well being of the patient.” “I think it is important to mention family-centered care in undergraduate and graduate schooling when it comes to healthcare professions. I do not think it is mentioned enough, especially for those who are planning on working in/around the secondary school setting.” “More education and continuing education programs furthering the need for enhancement a family centered care in the athletic training realm.”
**Staffing and space concerns**	“Funding and space for athletic training facilities in the secondary school setting that facilitate privacy, approachability and effectiveness of care rendered. Also, appropriate staffing schedule/number of personnel available in the secondary school setting in order to competently address the issues involved in family centered care from all aspects including documentation and in person care.” “Staffing needs to be increased. Athletic training rooms need to be redesigned.” “An increase in properly trained staff that are available to meet the needs of the students and not just during regular school hours. Staff need to be available before and after school as well.” “Double our staff in order to have time to be able to truly communicate with all necessary parties.” “Family-centered care is difficult in a secondary school setting that is very open in which most events occur at the same time. After school it gets very busy and there is not the time or privacy to have family-centered care. I worry about privacy in speaking to so many people about what my one athlete is going through.” “I am a solo athletic trainer in a large high school. I try to do all of these things, but some days I’m just able to keep everyone alive. Family centered care is important, but meeting all of these standards might not be possible for one athletic trainer.” “I think family centered care at its core is a valuable model for treating patients, especially those in the secondary school setting where they are bridging the gap between childhood and adult hood. However the logistics of effectively providing this type of care in a secondary school are unreasonable. At a school with roughly 1400 athletes, there is not enough time in the day to meet the needs of the patient, provide event coverage, and ensure the family is supported. In theory this would be a good model, however we do not have the staff or the resources to implement this in the secondary school.”
**Non-technical skills**	“Keeping open lines of communication between the child/athlete, family, and any other staff that may be involved in a case is key to ensure quality care. Some suggestions and ideas may not be suitable depending on the facility or protocols within a school’s district. This is something my coworkers and I have ran into within our district. We actively try to get involved in meetings when protocols and procedures are being reviewed to push for certain care initiatives.” “There are many obstacles in providing family centered care. In dealing with adolescents the majority of our parents want the child to take responsibility for their own care and are not involved in the care process. There are a small percentages of times where it is difficult to contact parent <2% of time. On the converse there are some parents who are too involved and do not see the athletic trainer as a healthcare professional and want their child to return to play even if the child is not ready. It is difficult to include the family many times due time constraints and parents not being available to contact as they are not physically at the school or at work and contacting via phone is often difficult.” “Enhance interdisciplinary care across care continuum” “Parents have the right to know (if the student is under 18) but siblings? Absolutely not. I even get worried when teachers and advisors learn of injuries or diagnosis prior to the medical staff at the school learning of it. There is a fine line between helping a student and the student not trusting us because information is too freely shared amongst everyone on campus.” “Trust between provider and families/parents are a huge asset toward quality and through care.”
**Social determinants of health**	“Additional resources to assist lower socioeconomic families whos medicare does not get accepted by certain medical providers (e.g., physical therapy clinics).” “Family centered care also depends on the area one works in-middle class, lower class, etc. I work with kids who come from low income families and needs are different.” “Family-centered care should include recommendations for grouping…For families within low socioeconomic status, for boarding students vs. day students, for children with rotating guardians.” “One of the biggest flaws I have in dealing with families is insurance, lack of insurance, and restrictions from insurance companies. Many of my student/athletes are from broken homes and neither parent wants financial responsibility.”

## Data Availability

The data presented in this study are available on request from the corresponding author. The data are not publicly available due to IRB protections.

## References

[1] Barry M.J., Edgman-Levitan S. (2012). Shared decision making—The pinnacle patient-centered care. N. Engl. J. Med..

[2] Wolf D.M., Lehman L., Quinlin R., Zullo T., Hoffman L. (2008). Effect of patient-centered care on patient satisfaction and quality of care. J. Nurs. Care Qual..

[3] Board of Certification for the Athletic Trainer Content Outline for Practice Analysis, 8th edition. https://bocatc.org/system/document_versions/versions/301/original/boc-pa8-content-outline-20230109.pdf?1673284659.

[4] Redinger A.S., Winkelmann Z.K., Eberman L.E. (2021). Collegiate student-athletes’ perceptions of patient-centered care delivered by athletic trainers. J. Athl. Train..

[5] David S., Larson M. (2018). Athletes’ perception of athletic trainer empathy: How important is it?. J. Sport Rehab..

[6] Kahanov L., Fairchild P.C. (1994). Discrepancies in perceptions held by injured athletes and athletic trainers during the initial injury evaluation. J. Athl. Train..

[7] Courson R., Goldenberg M., Adams K.G., Anderson S.A., Colgate B., Cooper L., Dewald L., Floyd R.T., Gregory D.B., Indelicato P.A. (2014). Inter-association consensus statement on best practices for sports medicine management for secondary schools and colleges. J. Athl. Train..

[8] Adado S.R., Games K.E. (2021). Parental Perceptions of the Importance and Effectiveness of Patient-Centered Care Delivery. Int. J. Athl. Ther. Train..

[9] Kuo D.Z., Houtrow A.J., Arango P., Kuhlthau K.A., Simmons J.M., Neff J.M. (2012). Family-centered care: Current applications and future directions in pediatric health care. Matern. Child Health. J..

[10] Foster K., Young A., Mitchell R., Van C., Curtis K. (2017). Experiences and needs of parents of critically injured children during the acute hospital phase: A qualitative investigation. Injury.

[11] Pike Lacy A.M., Eason C.M., Chu E.L., Stearns R.L., Casa D.J. (2022). Youth athletes’ parents’ perceptions and knowledge of the athletic training profession. J. Athl. Train..

[12] Coyne I., Holmström I., Söderbäck M. (2018). Centeredness in healthcare: A concept synthesis of family-centered care, person-centered care and child-centered care. J. Pediatr. Nurs..

[13] Kokorelias K.M., Gignac M.A., Naglie G., Cameron J.I. (2019). Towards a universal model of family centered care: A scoping review. BMC Health Serv. Res..

[14] Bruce B., Ritchie J. (1997). Nurses’ practices and perceptions of family-centered care. J. Pediatr. Nurs..

[15] Bruce B. (1992). Nurses’ Perceptions and Practices of Family-Centred Care. Master’s Thesis.

[16] Polit D.F., Beck C.T. (2006). The content validity index: Are you sure you know what’s being reported? Critique and recommendations. Res. Nurs. Health.

[17] Neal T.L., Diamond A.B., Goldman S., Klossner D., Morse E.D., Pajak D.E., Putukian M., Quandt E.F., Sullican J.P., Wallack C. (2013). Inter-association recommendations for developing a plan to recognize and refer student-athletes with psychological concerns at the collegiate level: An executive summary of a consensus statement. J. Athl. Train..

[18] Franck L.S., Cormier D.M., Hutchison J., Moore D., Bisgaard R., Gay C., Ngo S., Kriz R., Lin C., Ekno M. (2021). A multisite survey of NICU healthcare professionals’ perceptions about family-centered care. Adv. Neonatal Care.

[19] Caty S., Larocque S., Koren I. (2001). Family-centered care in Ontario general hospitals: The views of pediatric nurses. Can. J. Nurs. Leadersh..

[20] O’Neil M.E., Palisano R.J. (2000). Attitudes toward family-centered care and clinical decision making in early intervention among physical therapists. J. Pediatr. Phys. Ther..

[21] West J.J., Winkelmann Z.K., Edler J., Jackson B.C., Eberman L.E. (2020). adolescent perceptions of injury and pressures of returning to sport: A retrospective qualitative analysis. J. Sport. Med. Allied Health Sci. Off. J. Ohio Athl. Train. Assoc..

[22] Wilcoxon L.A., Meiser-Stedman R., Burgess A. (2021). Post-traumatic stress disorder in parents following their child’s single-event trauma: A meta-analysis of prevalence rates and risk factor correlates. Clin. Child Fam. Psychol. Rev..

[23] Shelton T.L. (1987). Family-Centered Care for Children with Special Health Care Needs.

[24] Nathanson M.N. (1986). Organizing and Maintaining Support Groups for Parents of Children with Chronic Illness and Handicapping Conditions.

[25] Petersen M.F., Cohen J., Parsons V. (2004). Family-centered care: Do we practice what we preach?. J. Obstet. Gynecol. Neonatal Nurs..

[26] Phiri P.G., Chan C.W., Wong C.L., Choi K.C., Ng M.S. (2022). Discrepancies between nurses’ current and perceived necessary practices of family-centred care for hospitalised children and their families: A cross-sectional study. J. Pediatr. Nurs..

[27] Bruce B., Letourneau N., Ritchie J., Larocque S., Dennis C., Elliott M.R. (2002). A multisite study of health professionals’ perceptions and practices of family-centered care. J. Fam. Nurs..

[28] Coyne I., Murphy M., Costello T., O’Neill C., Donnellan C. (2013). A survey of nurses’ practices and perceptions of family-centered care in Ireland. J. Fam. Nurs..

[29] Prasopkittikun T., Srichantaranit A., Chunyasing S. (2020). Thai nurses’ perceptions and practices of family-centered care: The implementation gap. Int. J. Nurs. Sci..

[30] Alabdulaziz H., Moss C., Copnell B. (2017). Paediatric nurses’ perceptions and practices of family-centred care in Saudi hospitals: A mixed methods study. Int. J. Nurs. Stud..

[31] Smith M.A. (2016). The role of shared decision making in patient-centered care and orthopaedics. Orthop. Nurs..

[32] Stucki G., Cieza A., Ewert T., Kostanjsek N., Chatterji S., Ustun T.B. (2002). Application of the International Classification of Functioning, Disability and Health (ICF) in clinical practice. Disabil. Rehabil..

[33] Ross J.R., Capozzi J.D., Matava M.J. (2012). Discussing treatment options with a minor: The conflicts related to autonomy, beneficence, and paternalism. JBJS.

[34] Weitzel R.L., Miller M.G., Giannotta E.R., Newman C.J. (2015). High school athletes’ parents’ perceptions and knowledge of the skills and job requirements of the certified athletic trainer. J. Athl. Train..

[35] Pike Lacy A.M., Bowman T.G., Huggins R.A., Lininger M.R., Denegar C.R., Casa D.J., Singe S.M. (2022). Secondary school athletic trainers’ experiences with organizational conflict: A comparison across employment models. J. Athl. Train..

[36] Giordano G.N., Merlo J., Ohlsson H., Rosvall M., Lindström M. (2013). Testing the association between social capital and health over time: A family-based design. BMC Public Health.

[37] Lawlor M.C., Mattingly C.F. (1998). The complexities embedded in family-centered care. Am. J. Occup. Ther..

[38] Macdonald I., Hauber R. (2016). Educating parents on sports-related concussions. J. Neurosci. Nurs..

[39] Yin H.S., Dreyer B.P., Vivar K.L., MacFarland S., van Schaick L., Mendelsohn A.L. (2012). Perceived barriers to care and attitudes towards shared decision-making among low socioeconomic status parents: Role of health literacy. Acad. Pediatr..

[40] McGuine T.A., Pfaller A.Y., Post E.G., Hetzel S.J., Brooks A., Broglio S.P. (2018). The influence of athletic trainers on the incidence and management of concussions in high school athletes. J. Athl. Train..

[41] Mazerolle S.M., Raso S.R., Pagnotta K.D., Stearns R.L., Casa D.J. (2015). Athletic directors’ barriers to hiring athletic trainers in high schools. J. Athl. Train..

[42] Huggins R.A., Coleman K.A., Attanasio S.M., Cooper G.L., Endres B.D., Harper R.C., Huemme K.L., Morris R.F., Pike Lacy A.M., Peterson B.C. (2019). Athletic trainer services in the secondary school setting: The athletic training locations and services project. J. Athl. Train..

[43] Walker S.E., Pitney W.A., Lauber C.A., Berry D. (2008). An exploration of athletic trainers’ perceptions of the continuing education process. Internet J. Allied Health Sci. Pract..

[44] Armstrong K.J., Weidner T.G. (2011). Preferences for and barriers to formal and informal athletic training continuing education activities. J. Athl. Train..

[45] Edler J.R., Eberman L.E. (2019). Factors influencing athletic trainers’ professional development through continuing education. Athl. Train. Educ. J..

[46] Philibert I., Patow C., Cichon J. (2011). Incorporating patient-and family-centered care into resident education: Approaches, benefits, and challenges. J. Grad. Med. Educ..

[47] Welch Bacon C.E., Anderson B.E., Cavallario J.M., Van Lunen B.L., Eberman L.E. (2022). Content validation of the athletic training milestones: A report from the AATE research network. J. Athl. Train..

[48] (2020). The Accreditation Council for Graduate Medical Education Internal Medicine Milestones. https://www.acgme.org/globalassets/pdfs/milestones/internalmedicinemilestones.pdf.

[49] Jaquith S., Hanley M. (2018). Parents’ Perception of Athletic Trainers in the High School Setting: Sacred Heart University. https://digitalcommons.sacredheart.edu/acadfest/2018/all/108/.

[50] Picha K.J., Welch Bacon C.E., Normore C., Snyder Valier A.R. (2022). Social determinants of health: Considerations for athletic health care. J. Athl. Train..

[51] Hernandez M.I., Miller E.C., Biese K.M., Columna L., Andreae S.J., McGuine T., Snedden T., Eberman L., Bell D.R. (2022). Secondary School Athletic Trainers’ Navigation of Patient Socioeconomic Status Challenges in Care: A Qualitative Study. Int. J. Environ. Res. Public Health.

[52] Giorgi E.M., Drescher M.J., Winkelmann Z.K., Eberman L.E. (2022). Validation of a script to facilitate social determinant of health conversations with adolescent patients. Int. J. Environ. Res. Public Health.

